# Improving Engagement in Journal Club: Integration of MRCPsych-Style MCQs Into the Local Academic Programme in Mersey Care NHS Foundation Trust

**DOI:** 10.1192/bjo.2026.11389

**Published:** 2026-06-30

**Authors:** Ann Gollo, Mahmoud Abdelaal, Indira Vinjamuri

**Affiliations:** Mersey Care NHSFT, Liverpool, United Kingdom

## Abstract

**Aims:**

Journal Club Presentations (JCPs) are a mandatory component of the Royal College of Psychiatrists (RCPsych) portfolio requirements for Annual Review of Competence Progression (ARCP), with trainees required to complete at least one JCP per training year. At Mersey Care NHS Foundation Trust, JCPs are delivered within the weekly Local Academic Programme (LAP), but trainees frequently report reduced engagement, particularly during statistical discussions. Baseline LAP feedback identified difficulty understanding statistics, limited interactivity, and low perceived examination relevance as key contributors. As critical appraisal and research methods form a substantial component of the MRCPsych Paper B examination, this Quality Improvement Project aimed to enhance engagement by integratingbrief MRCPsych-style multiple-choice questions (MCQs), targeting a 25% improvement from baseline.

**Methods:**

This prospective quality improvement project used a pre-post descriptive design within the weekly LAP. Baseline data were obtained from routinely collected LAP feedback surveys (August–September 2025; pooled n=427), assessing engagement, barriers to participation, and acceptability of MCQs. From 16 September 2025, MRCPsych-style MCQs were incorporated into every JCP. Presenters were asked to include 1–3 MCQs focused on statistical or methodological concepts relevant to the paper, taking no more than five minutes. Post-intervention evaluation was conducted at two predefined time points in December 2025 (pooled n=211). Outcomes included self-reported engagement on a 10-point Likert scale, understanding of journal articles, perceived knowledge retention, and interactivity. Data were analysed descriptively.

**Results:**

At baseline, 25–30% of respondents rated engagement as average, 40–50% reported difficulty understanding statistics, and 30–40% requested greater interactivity. Post-intervention, 65–70% reported a moderate to very significant increase in engagement (≥7/10), exceeding the 25% improvement target. Improved understanding of journal articles was reported by 63–65%, with up to 39.5% reporting better statistical understanding and overall comprehension. Knowledge retention improved for 69–70%, and interactivity for approximately 67%. Only 3–6% reported minimal or no improvement. Qualitative feedback was strongly positive, with requests to continue and expand MCQ use.

**Conclusion:**

Sustained integration of MRCPsych-style MCQs into JCPs was associated withmeaningful improvements in engagement, understanding, retention, and interactivity. This lowcost, scalable intervention exceeded its primary objective and has now been embedded into routine teaching practice. Incorporating brief, exam-relevant interactive elements represents an effective strategy for enhancing postgraduate psychiatric education.

